# Implicit Incentives Among Reddit Users to Prioritize Attention Over Privacy and Reveal Their Faces When Discussing Direct-to-Consumer Genetic Test Results: Topic and Attention Analysis

**DOI:** 10.2196/35702

**Published:** 2022-08-03

**Authors:** Yongtai Liu, Zhijun Yin, Zhiyu Wan, Chao Yan, Weiyi Xia, Congning Ni, Ellen Wright Clayton, Yevgeniy Vorobeychik, Murat Kantarcioglu, Bradley A Malin

**Affiliations:** 1 Department of Computer Science Vanderbilt University Nashville, TN United States; 2 Department of Biomedical Informatics Vanderbilt University Medical Center Nashville, TN United States; 3 School of Law, Vanderbilt University Nashville, TN United States; 4 Department of Pediatrics, Vanderbilt University Medical Center Nashville, TN United States; 5 Department of Health Policy Vanderbilt University Medical Center Nashville, TN United States; 6 Department of Computer Science and Engineering, Washington University in St. Louis St. Louis, MO United States; 7 Department of Computer Science, University of Texas at Dallas Richardson, TX United States; 8 Department of Biostatistics Vanderbilt University Medical Center Nashville, TN United States

**Keywords:** direct-to-consumer genetic testing, topic modeling, social media

## Abstract

**Background:**

As direct-to-consumer genetic testing services have grown in popularity, the public has increasingly relied upon online forums to discuss and share their test results. Initially, users did so anonymously, but more recently, they have included face images when discussing their results. Various studies have shown that sharing images on social media tends to elicit more replies. However, users who do this forgo their privacy. When these images truthfully represent a user, they have the potential to disclose that user’s identity.

**Objective:**

This study investigates the face image sharing behavior of direct-to-consumer genetic testing users in an online environment to determine if there exists an association between face image sharing and the attention received from other users.

**Methods:**

This study focused on r/23andme, a subreddit dedicated to discussing direct-to-consumer genetic testing results and their implications. We applied natural language processing to infer the themes associated with posts that included a face image. We applied a regression analysis to characterize the association between the attention that a post received, in terms of the number of comments, the karma score (defined as the number of upvotes minus the number of downvotes), and whether the post contained a face image.

**Results:**

We collected over 15,000 posts from the r/23andme subreddit, published between 2012 and 2020. Face image posting began in late 2019 and grew rapidly, with over 800 individuals revealing their faces by early 2020. The topics in posts including a face were primarily about sharing, discussing ancestry composition, or sharing family reunion photos with relatives discovered via direct-to-consumer genetic testing. On average, posts including a face image received 60% (5/8) more comments and had karma scores 2.4 times higher than other posts.

**Conclusions:**

Direct-to-consumer genetic testing consumers in the r/23andme subreddit are increasingly posting face images and testing reports on social platforms. The association between face image posting and a greater level of attention suggests that people are forgoing their privacy in exchange for attention from others. To mitigate this risk, platform organizers and moderators could inform users about the risk of posting face images in a direct, explicit manner to make it clear that their privacy may be compromised if personal images are shared.

## Introduction

The cost of genome sequencing has steadily decreased over time [1], which, in turn, has enabled the emergence of direct-to-consumer genetic testing (DTC-GT) services available to the public [2]. DTC-GT allows consumers to learn about their genetic information without consulting with a health care provider [3]. The number of people who have participated in DTC-GT has increased dramatically, growing from 12 million in January 2018 to 26 million in January 2019 [4]. As of late 2021, the two largest DTC-GT companies, AncestryDNA and 23andme, had amassed over 20 million and 12 million clients, respectively [5]. Recent studies indicate that people pursue DTC-GT for various reasons, primarily to learn about their ancestry and to discover or confirm kinship [6,7].

As DTC-GT services have grown in popularity, consumers have increasingly relied upon online social platforms to discuss and share their test results (though not always the raw genome sequences) [8]. One particularly notable platform is Reddit, an online content rating and discussion site where users can create different subreddits based on specific topics of interest. One of the most popular subreddits related to DTC-GT is r/23andme, with more than 81,400 subscribers as of May 2022. In r/23andme, users discuss a wide range of topics related to genetic testing, including testing services, test results, explanations and interpretations, and share stories about what happened after undergoing testing (eg, health-related decisions) [8].

When r/23andme users share their results for discussion, instead of simply typing text, some users attach a screenshot of their DTC-GT result page (eg, the ancestry composition). Since Reddit is a virtual online community where users generally rely upon pseudonyms for communication, such screenshots of results typically do not contain a user’s real name. Therefore, even when users share and discuss their DNA test results, this subreddit has historically been a community with a culture of anonymity.

**Figure 1:**
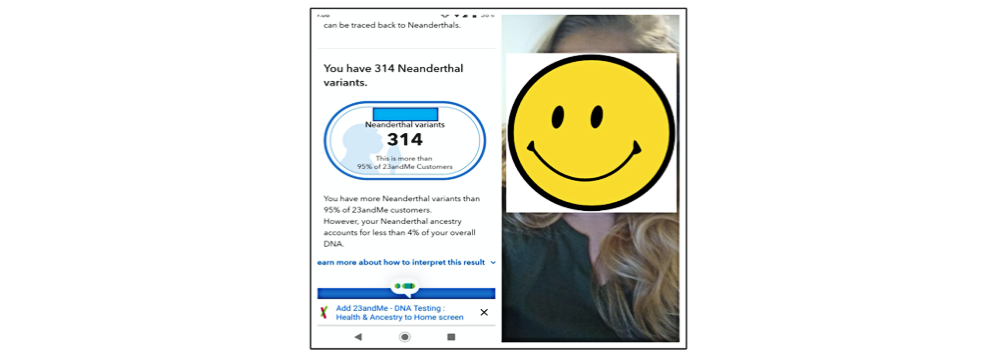
An example of a face image posted on the r/23andme subreddit. The report is shown together with a face image and testing results. The actual face and name are obscured for this publication; however, the data exist in the public domain.

However, in 2019, r/23andme users began attaching personal images to their posts. Figure 1 presents an example of a screenshot of a user’s DTC-GT result page on the left, with the full-face image of this user on the right. This movement toward revealing one’s face directly affects personal privacy [9,10]. Although these posts used pseudonyms, face image posting in online environments constitutes a knowing decision to give up one’s privacy. Other users may utilize these face images to determine a user’s identity, relying, in part, on the rapid development and deployment of modern face recognition [11] and identity detection systems [12]. This is a concern, because identity disclosure may lead to various negative consequences for individuals, including identity theft [13], discrimination [14], and threats to personal safety [15]. Since Reddit is a public platform, a user’s posts and face images are readily accessible, making an identity disclosure attack feasible with little cost [16].

Though users may be aware that revealing their face likely compromises their privacy, it is unclear why they choose to do so. Various investigations into behavioral psychology and economics show that some people waive their privacy rights in exchange for a service that they value [17]. Thus, we hypothesize that r/23andme users may receive more attention by publishing more personal information. This is supported by findings on other social platforms. For instance, including photos with tweets on the Twitter platform can boost retweets by 35% [18]. Instagram photos with faces are 38% more likely to receive likes and 32% more likely to receive comments [19]. However, unlike Twitter or Instagram, the DTC-GT forum examined in this paper provides an anonymous environment for users to share and discuss sensitive personal genetic information. Thus, we sought to determine whether this forum supports the same privacy-service exchange hypothesis. To formally test our hypothesis, we investigated the following research questions: (1) What are the topics communicated in the natural language of posts with face images? (2) Is face image posting associated with the attention that a post receives?

To answer these questions, we collected posts from the r/23andme subreddit and categorized them into three types: (1) posts with only text, (2) posts with face images, and (3) posts with images not containing a face. We next measured the temporal posting trends regarding the type of post. Then, we applied topic modeling to compare the primary topics associated with types of post. Finally, we performed a regression analysis to infer the association between the attention that a post received, in terms of votes, comments, and whether the post contained a face image.

## Methods

### Ethics Considerations

This study involved only online posts that were openly accessible on Reddit. We have published the analysis results only in this paper, and any referenced posts or figures have been anonymized to protect the privacy of users.

### Overview

**Figure 2:**
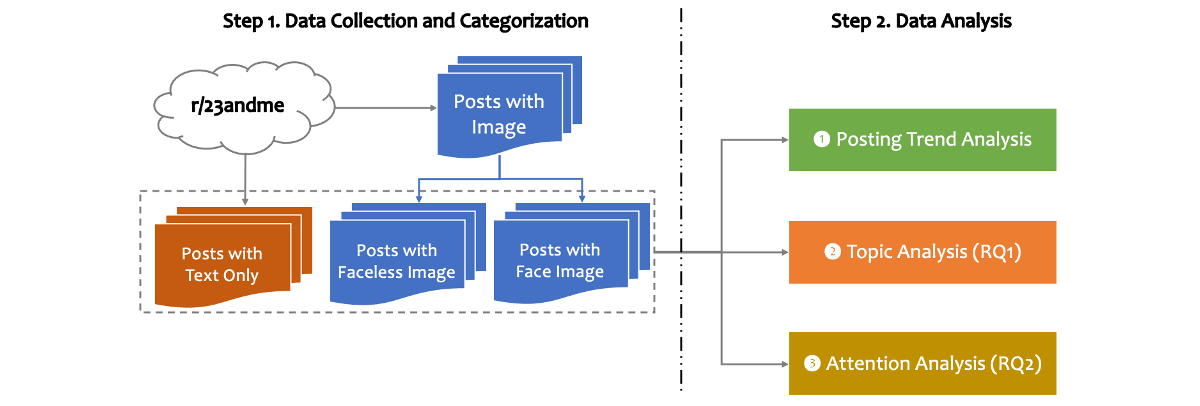
An overview of the research workflow for r/23andme post analysis. RQ: research question.

Figure 2 provides an overview of the research pipeline, which had two primary steps. The first step involved data collection and categorization, in which we collected the posts on the r/23andme subreddit and extracted those with a face image using face recognition software. The second step focused on analysis. Specifically, we first conducted an exploratory analysis to investigate the temporal posting trends and then leveraged topic modeling to infer the themes communicated in these posts. Finally, we performed a regression analysis to determine whether including a face image in a post was associated with the attention it received. In this study, we characterized attention by the number of comments and the karma score that a post received from other online users. The karma score on Reddit is defined as the number of upvotes minus the number of downvotes, indicating the popularity of a post.

### Data Collection and Categorization

To collect data from the r/23andme subreddit, we first gathered the IDs of all posts (ie, submissions) and comments using pushshift.io. We then applied the Python Reddit application programming interface wrapper package (version 6.3.1) to extract data from Reddit for each post ID. Specifically, we collected all posts and comments published on r/23andme between December 31, 2012, and January 31, 2020. Each collected post contained the following information: (1) author identifier, (2) post title, (3) post text body, (4) image URL (if there was an image in the post), (5) comments on the post, (6) post date, and (7) karma scores of the post and affiliated comments.

We downloaded the images from posts containing an image URL and applied the face-recognition Python package (version 1.3.0) [20] to classify images into (1) images with a face and (2) images without a face (ie, faceless images). To assess the accuracy of the face detection algorithm, we randomly selected 100 images from each group and manually examined the quality of classification. We found that 7 faceless images were classified as face images, indicating a false positive rate of 7% (7/100), while 2 face images were classified as faceless images, indicating a false negative rate of 2% (2/100). To achieve 100% precision, we manually reviewed all the images in the face group and relabeled the misclassified images. Due to a high true positive rate of 98% (98/100) and the large volume of the faceless images (3865), we did not perform a manual review step for the set of faceless images. As such, we categorized all of the collected posts into three types: (1) text-only posts; (2) posts with faceless images; and (3) posts with face images (such as the post in Figure 1), corresponding to 3 types of users.

### Data Analysis

To describe face image posting behavior, we compared the face posts with the other two types of posts along three perspectives: (1) posting temporal trend, (2) post theme, and (3) the attention that a post received from other users, in terms of the number of comments and karma score.

#### Topic Analysis

To examine the thematic differences between the three post types, we applied topic modeling [21] to the post title rather than the post body, because 41.1% (6404/15,596) of the posts had an empty text body. We first tokenized the data and removed all punctuation. Next, we lemmatized words into their base forms (eg, “walks” became “walk”) using the nltk Python package (version 3.3). We also replaced personal pronouns, such as *“*we,” “she,” and “they,” with the symbol “-PRON-,” and replaced numbers with the word “datum.” We then applied latent Dirichlet allocation (LDA) [22], as implemented in the gensim Python package (version 3.8.1), to extract topics. Since LDA is an unsupervised learning model, we calibrated the number of topics for the optimal model based on the coherence score, which measures the pairwise word semantic similarity in a topic. To do so, we ran LDA models with 2 to 20 topics (using a step size of 2) on the set of lemmatized words and selected the topic number that achieved the highest coherence score. Finally, to demonstrate the quality of topic modeling, we used *t*-distributed stochastic neighbor embedding [23] to cluster topics and displayed the results as a 2D representation (Figure S1 and Figure S2 in Multimedia Appendix 1).

#### Regression Analysis

We investigated two types of associations. First, we considered the association between an image post (with and without a face) and the attention it received. Second, we considered the association between a face post and the attention it received. Since the number of comments and the karma score are nonnegative count variables, we applied a negative binomial regression to infer the association [24].

Given that posts published earlier may be read by more readers and, thus, receive more comments and votes, we included the number of days a post had been published as a control variable. In addition, posts on different topics might receive different levels of attention. To reduce the effects of post topic, we incorporated the topic distribution of each post as an additional set of control variables. During model fitting, we dropped one topic (T_4_, see below) to address collinearity.

Moreover, the activity level of users might affect the popularity of their posts. For example, posts from active users may receive more attention. To reduce the impact of user activity, we incorporated the number of posts and the number of comments of each user as an additional set of control variables. We utilized the implementation of negative binomial regression in the statsmodels Python package (version 0.11.1) to fit models for the karma score and the number of comments separately. We reported the features that achieved statistical significance at the *P*<.001 level.

## Results

We collected 15,596 posts and 188,843 comments, which were published by 20,883 users between December 31, 2012, and January 31, 2020. Among the collected posts, 24.8% (3818/15,596) contained faceless images, while 5.4% (849/15,596) contained face images.

### Temporal Trends

**Figure 3:**
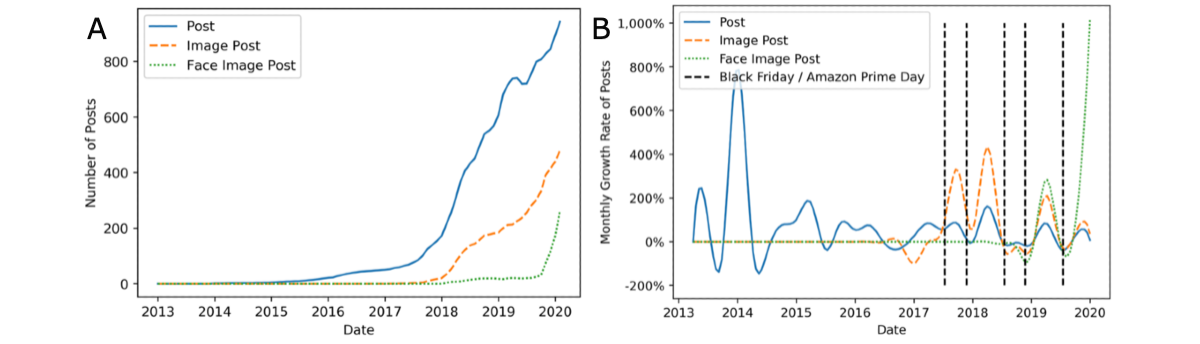
Smoothed temporal trends of three types of post, including the number of posts published per month (A) and quarterly growth rate of posts (B).

In Figure 3A, the graph depicts the temporal post trend on a monthly basis. It can be seen that the r/23andme subreddit exhibited relatively low activity until 2017, after which the number of monthly posts grew rapidly. Image posts (with and without a face) became popular after 2018. In Figure 3B, the graph shows the quarterly growth rate of the number of posts. The green dotted line indicates that, since 2019, the number of face posts exhibited a rapid increase, with a growth rate that surpassed the growth rate of all posts (represented by the blue line) and image posts (represented by the orange dashed line). Notably, we find that posting rates for all 3 types of post increased rapidly after major promotions by 23andme (eg, as part of Black Friday and Amazon Prime Day, advertising events held by Amazon Inc), which is consistent with the findings of Yin et al [8].

### Attention to Posts

**Figure 4:**
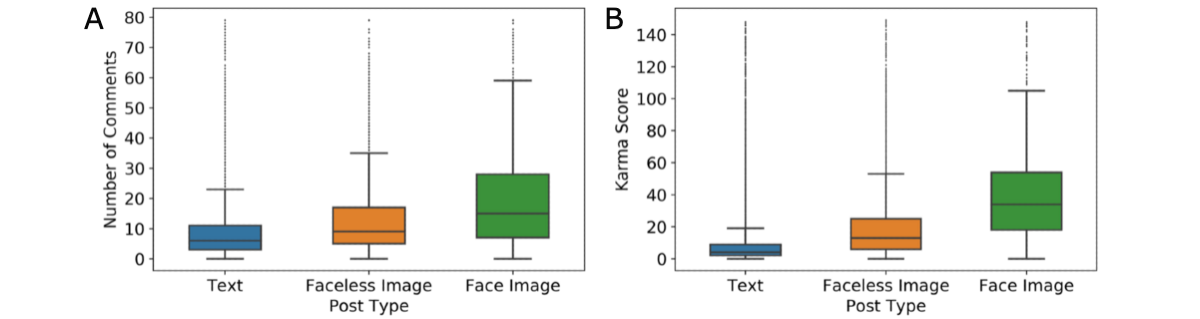
Attention to three types of posts. The number of comments per post (A) and karma score per post (B). For presentation purposes, we removed posts with more than 80 comments or karma scores greater than 150 (3% of the data). The entire data set is provided in Figure S3 and Figure S4 in Multimedia Appendix 1.

Figure 4A is a boxplot showing the number of comments per post for each post type. Face posts received the most comments, followed by posts not containing a face. The median number of comments for text-only posts was 6, but the median increased to 9 for posts with faceless images and 13 for posts with face images. Figure 4B is a boxplot showing the karma score by post type. Face posts received the highest median karma score (34), followed by posts with faceless images (median karma score 13). In contrast, the median karma score for text posts was only 4. One-way ANOVA tests for comments and karma scores indicated that the differences were statistically significant (*P*<.001).

### User Activity

**Figure 5:**
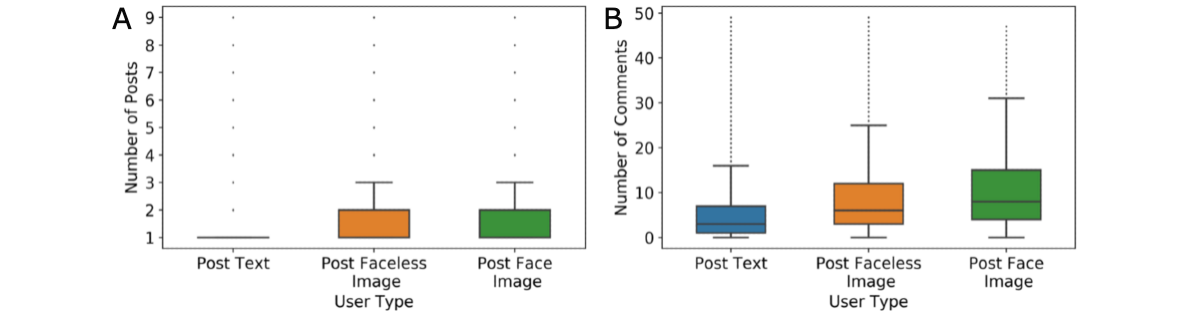
Number of posts per user (A) and number of comments per user (B) for users who posted (1) text only, (2) faceless images, and (3) face images. For presentation purposes, we removed users who published more than 10 posts or 50 comments, accounting for 4.4% of the total number of users. The entire data set is provided in Figure S3 and Figure S4 in Multimedia Appendix 1.

We measured user activity in terms of the number of posts and comments. We found that 26.8% (2442/9114) of the users posted faceless images, while 8.5% (774/9114) posted face images. Figure 5A is a graph showing that the median number of posts for all 3 user types was 1. However, the third quartile of users who posted images (with or without a face) was 2. This suggests that, on average, authors who posted images (with or without a face) had more posts than authors who posted only text. The graph in Figure 5B depicts the number of comments posted for each user type. The users who posted face images wrote the most comments, with a median of 8. The median dropped to 6 for users who posted images not containing a face. For users who posted only text, the median number of comments was substantially lower, at 3. The results of 1-way ANOVA tests for the number of posts and the number of comments indicated that the differences were statistically significant (*P*<.001).

### Topic Analysis

Table 1 shows the 10 inferred topics, their most relevant words, and the topic distribution (Figure S1 and Figure S2 in Multimedia Appendix 1 show details on the selection of the number of topics). The most relevant words were ranked based on their marginal distribution within a topic and displayed in descending order. The topic distribution was calculated as the percentage of posts belonging to the topic. Based on the relevant words and posts with the highest probability for each topic, we further grouped the 10 topics into three categories: (1) ancestry composition, (2) kinship and family discovery, and (3) general questions about genetic testing.

Ancestry composition included 4 topics: T_1_, T_2_, T_3_, and T_4_. Posts in this category focused on the presentation and discussion of ancestry composition testing results. The 4 topics captured ancestry information, which communicate a user’s race, continental origin, and nationality. Textbox 1 shows example posts for each topic. Kinship finding and family discovery was communicated in T_5_ and T_6_. Specifically, T_5_ communicated the discovery of ancestors and distinct relatives, where it can be seen that terms like “family” and “history” were often used. In T_6_, words such as “find,” “dad,” and “siblings” show that this topic focused on findings relating to immediate family members. General questions related to DTC-GT were communicated in T_7_, T_8_, T_9_, and T_10_. Specifically, T_7_ posts mainly asked about testing service progress. Words such as “time” and “wait” were highly weighted in this topic. T_8_ posts were mainly comparisons of DTC-GT companies. There were mentions of companies, such as “MyHeritage,” “23andme,” and “WeGene.” T_9_ covered posts about understanding, or questions about, the test result report. T_10_ posts mainly discussed an upgrade to the genetic testing algorithm and the subsequent changes in testing results. Words such as “beta,” “update,” and “change” were highly weighted.

**Figure 6:**
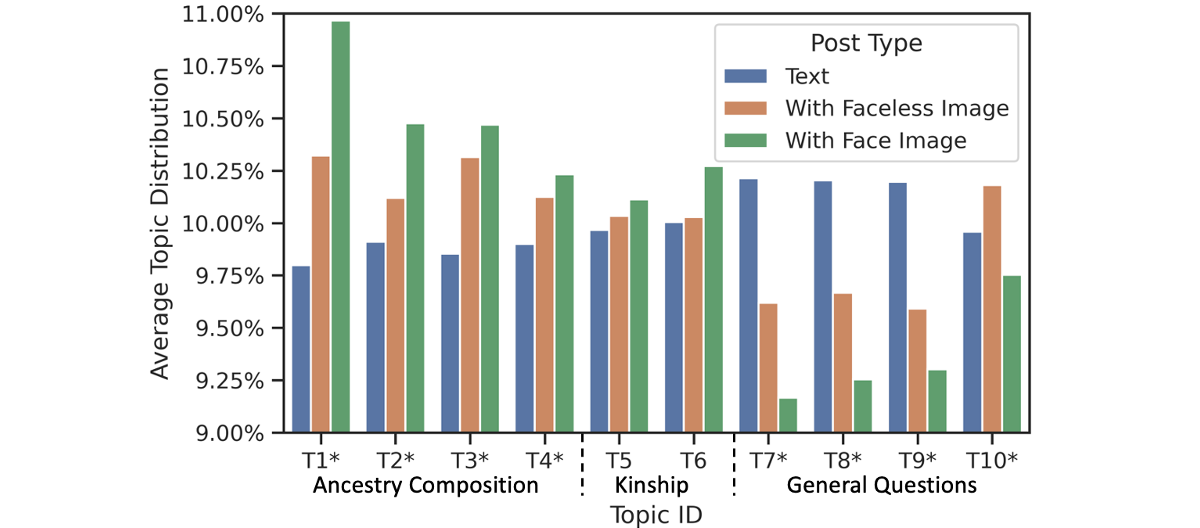
The prevalence of topics for each post type. The topics are arranged according to category. *P<.001 according to a 1-way ANOVA with post-hoc Tukey honestly significant difference tests for pairwise differences between the 3 post types for the topic.

Figure 6 presents the topic distribution for each type of post. The 1-way ANOVA tests showed that there were statistically significant differences between the means of the 3 post types for all 10 topics (*P*<.001). Face posts were more likely to communicate ancestry composition (T_1_, T_2_, T_3_, and T_4_) and kinship and family discovery (T_5_ and T_6_), while text posts were more likely to be about general questions (T_7_, T_8_, and T_9_). T_10_, a topic about an algorithm upgrade by 23andMe, shows that faceless image posts were more likely to communicate this topic, followed by text posts and then face image posts. This may be because users tended to post screenshots of the results before and after the algorithm upgrade for easy comparison.

**Table 1:** The topics inferred from the r/23andme subreddit. The sample words are presented in descending order according to their relevance score within the topic.

Category		Top-20 most relevant terms	Topic distribution	
**Ancestry composition**				
	Topic 1	European, -PRON-, result, Italian, Irish, British, surprise, Jewish, white, Chinese, broadly, bit, eastern, Ashkenazi, surprised, Scandinavian, give, eye, lot, surprising		11.6%
	Topic 2	-PRON-, ancestry, German, guess, French, make, post, heritage, year, ethnicity, grandmother, common, grandparent, explain, mega-thread, feel, polish, Canadian, confused, wrong		7.9%
	Topic 3	result, -PRON-, expect, finally, back, ancestor, interesting, pretty, AncestryDNA, bear, confidence, recent, location, Filipino, cool, guy, live, thought, Finnish, big		9.1%
	Topic 4	American, Asian, African, native, Mexican, people, south, percentage, region, Neanderthal, gene, high, part, Spanish, unassigned, east, north, variant, trace, add		10.6%
**Kinship and family discovery**				
	Topic 5	-PRON-, family, today, close, tree, understand, worth, info, don, trait, history, link, happen, picture, excited, love, list, connection, inherit, risk		6.5%
	Topic 6	-PRON-, find, dad, half, mom, father, cousin, mother, side, sister, adopt, brother, great, sibling, grandfather, full, grandma, biological, aunt, figure		9.2%
**General questions**				
	Topic 7	kit, long, time, extraction, wait, timeline, genetic, day, receive, sample, analysis, week, testing, step, send, batch, fail, information, work, stick		14.2%
	Topic 8	andme, ancestry, datum, health, raw, accurate, GEDmatch, MyHeritage, good, DNA, upload, compare, site, comparison, land, data, service, difference, WeGene, interpret		11.0%
	Topic 9	DNA, test, relative, question, parent, report, share, -PRON-, phase, show, generation, relate, computation, person, unexpected, noise, mystery, relationship, account, number		9.7%
	Topic 10	result, update, beta, haplogroup, match, maternal, change, paternal, chromosome, map, mixed, chip, Puerto Rican, Korean, lose, comment, late, original, Romanian		10.2%

**Examples of posts for different topics.**

- “So I’m a lot less British than I thought, and a lot more Swiss” (Topic 1).
- “Any guesses on my friend’s ethnicity? He thinks he’s French/German, English, and maybe some Slavic” (Topic 2).
- “Born and raised in Manila, grew up thinking I was 100% Filipino. A bit shocked at my results” (Topic 3).
- “Found out I am East Asian and Native American but I have northern Asian and Native American so high” (Topic 4).
- “Found out I have about a dozen cousins I didn’t know about” (Topic 6).
- “My cousin did the DNA test and connected us to our great grandmother’s family!” (Topic 5).
- “On my account apparently my mom and her twin sister are both my moms” (Topic 6).
- “Is my kit moving slow? It took 2 weeks to be marked as “arrived” after tracking showed it was delivered” (Topic 7).
- “23andMe vs WEGENE – uploaded 23andMe raw data to WEGENE and here are the differences” (Topic 8).
- “What is a likely relationship if the shared DNA is 1610 centimorgans across 80 segments?” (Topic 9).
- “Beta update v5.2 should now be available to all earlier chip (pre-V5) users, when opting into the Beta program” (Topic 10).

### Regression Analysis

Table 2 summarizes the results of the negative binomial regressions. *R* for image→comment and *R* for image→score indicate the association between the number of comments, karma score, and whether the post contained images, either faceless or with a face. Image posting exhibited statistically significant positive associations with both dependent variables, suggesting that image posts received more attention than text-only posts.

With respect to the *R* for face→comment and *R* for face→score tests, we selected 4717 image posts and assessed the association between the number of comments, karma score, and whether the image contained a face. Face image posting exhibited statistically significant positive associations with both dependent variables, which indicates that face posts received more attention than faceless posts. Comparing the *R* for image→comment and *R* for face→comment tests showed that posting a face image achieved a more positive impact on receiving comments. Comparing the *R* for image→score and *R* for face→score tests showed a similar result.

In addition, there were two notable findings with respect to the control variables. First, the log-transformed number of published days exhibited a negative association in the *R* for image→comment and *R* for image→score tests (*β*=–.09 for image→comment, *β*=–.26 for image→score, *P*<.001). Second, T_8_ (the DTC-GT company comparison) had a negative association in all 4 tests (*P*<.001 for image→comment and face→comment, *P*=.003 for image→score, and *P*=.013 for face→score), while topic T_7_ (asking about testing service progress) showed a negative association in *R* for image→score, *R* for face→score, and *R* for face→comment tests (*P*<.001 for image→score, *P*=.003 for face→score, and *P*=.04 for face→comment). The negative association between topics T_7_, T_8_, and face posting reinforce our previous finding that the topics in posts including a face were less likely to correspond to a general question about DTC-GT.

**Table 2:** Results of the regression analysis relating post type to comments and karma score. All associations were statistically significant (*P*<.001).

Negative binomial regression	Dependent variable	Independent variable	*β*	Z	SD	*P* value
*R* for image→comment	Number of comments	Posting image	.152	6.41	0.024	<.001
*R* for image→score	Karma score	Posting image	.618	12.35	0.050	<.001
*R* for face→comment	Number of comments	Posting face image	.451	10.21	0.044	<.001
*R* for face→score	Karma score	Posting face image	.760	9.64	0.079	<.001

## Discussion

### Principal Findings

This investigation made several notable findings. First, consistent with previous studies on other social platforms [18,19], we observed that posts with face images in the r/23andme subreddit received more attention than other posts. It is possible that the increase in attention drove the disclosure of personal information in this online environment. However, this is only a conjecture, as our investigation was not designed to be a causal analysis. Regardless of the motivation for face image posting, it is evident that this behavior has rapidly grown within this subreddit.

Second, the 10 inferred topics from the titles of r/23andme posts appeared to fall into three categories. Posts in the first category, which covered 4 out of 10 topics, focused on discussing users’ ancestry composition. Notably, the topics in this category were associated with a higher rate of image and face image posting. It was further observed that users invoked their face images as proof (or counterexamples) of the genetic testing results. Posts about kinship and family member discovery exhibited a moderate rate of face image sharing. When inspecting posts in this category, posts such as “finally find my half-sister,” with a group photo of a reunion attached, were more prevalent than in other categories. Finally, posts asking general questions about genetic testing, which focused on comparisons between DTC-GT companies, the progress of testing result delivery, and upgrades to testing algorithms, exhibited the lowest rate of image sharing.

Third, counter to our expectation, we found that the number of days a post was published was negatively associated with a post’s attention. One possible explanation for this result is that Reddit archives posts older than 6 months and no longer allows commenting on them. Thus, the number of comments and votes was limited for earlier posts. We further noticed that the topic related to general questions was negatively correlated with attention to a post.

### Related Work

Natural language processing techniques have been applied to various health care applications [25]. Considering health care–related social media studies as an example, Liu et al [26] analyzed the association between weight loss progress and Reddit users’ online interactions; Klein et al [27] relied upon Twitter data to identify potential cases of COVID-19 in the United States; and Ni et al [28] compared the attitudes of users of 4 different social platforms toward the “gene-edited babies” event. For DTC-GT, most investigations have focused on consumer motivations [29], health implications [30], and ethical implications [31], with only a handful considering the disclosure of test reports over social platforms [8,32,33]. Most previous studies that used social media data focused solely on mining knowledge from text. In this study, by taking image posting into consideration, we assess the behavior of personal image sharing on this DTC-GT forum.

This paper analyzes the association between face image sharing and attention paid to posts in an online setting; this setting may incentivize users to sacrifice their privacy in exchange for the benefit of a social response. This observation, however, does not imply that attention is undesirable in all cases, as several studies have shown that social engagement is beneficial to an individual’s physical and mental health. For instance, in a large online breast cancer forum, Yin et al [34] found that the volume of online interchange was positively associated with patient treatment adherence. Pan et al [35] found that receiving replies could benefit online participants in depression forums. Naslund et al [36] analyzed the benefits and risks of using social media as a potentially viable platform for offering support intervention to persons with mental disorders. Thus, the perceived benefits an individual receives from a service typically outweigh the perceived privacy risks in the near term. Nevertheless, given that privacy concerns tend to be understood only later on [37], Reddit may wish to consider warning users about the potential negative consequences of their actions.

### Limitations

Despite our findings, there are certain limitations to this work, which we believe serve as opportunities for future research. First, the face recognition package had an estimated 2% false negative rate, which means that approximately 76 of the 3865 face images (2%) were likely wrongly labeled as faceless images. These misclassified images might have influenced the accuracy of our findings, although not their overall direction. Second, most topics inferred from topic modeling were interpretable and intuitive, but topic T_10_ was difficult to interpret. As shown in Table 1, sample words for T_10_ conveyed different kinds of information: “Puerto Rican” and “Korean” are related to ancestry composition, whereas “late” and “lost” are evidence of asking about delivery progress. In this respect, newer topic modeling techniques [38-40] or language model–based topic modeling (eg, top2vec [41] and BERTopic [42]) may provide better insights into the semantics of posts on social platforms. Importantly, however, the quality of individual topics had little effect on our main conclusion, since the regression analysis (using the topic distribution as control variable; Table 2) and ANOVA test (without topic distribution; Figure 4) yielded the same finding—a statistically significant association between face image sharing on r/23andme and user engagement.

### Conclusions

DTC-GT users are increasingly posting full-face images with their DTC-GT results on social platforms. In this study, we investigated the trend in this behavior in the r/23andme subreddit to obtain insight into potential underlying motivations. Our findings show that such behavior began in September 2019 and experienced rapid growth, with over 849 face-revealing posts by early 2020. Furthermore, our study suggests that posts including a face received, on average, 60% (5/8) more comments and 2.4 times higher karma scores than other posts. Posts that included face images were primarily about sharing and discussing ancestry composition and sharing family reunion photos with relatives discovered via DTC-GT. These findings verify our hypothesis that posting a personal image is associated with receiving more online attention, which is consistent with previous findings that people appear to be willing to give up their privacy (ie, their personal images) in exchange for a benefit (ie, attention from others). Based on this analysis, platform organizers and moderators might inform users about the risk of posting face images in a direct, explicit manner and make it clear that users’ privacy may be compromised if personal images are disclosed.

## Appendix

Multimedia Appendix 1 Supplementary materials.

## Abbreviations

DTC-GT: direct-to-consumer genetic testing
NLP: natural language processing
LDA: latent Dirichlet allocation
