# Supplementary material for: Implicit Incentives Among Reddit Users to Prioritize Attention Over Privacy and Reveal Their Faces When Discussing Direct-to-Consumer Genetic Test Results: Topic and Attention Analysis
Source: JMIR Infodemiology. 2022 Aug 3;2(2):e35702. doi: 10.2196/35702 (PMC9987181; doi:10.2196/35702)
Supplement: Multimedia Appendix 1 [file infodemiology_v2i2e35702_app1.docx]

Supplemental Material for “Prioritizing Attention over Privacy: Reddit Users are Implicitly Incentivized to Reveal Their Face When Discussing Their Direct-to-Consumer Genetic Test Results”

Yongtai Liu, MS^1^, Zhijun Yin, PhD^1,2^, Zhiyu Wan, PhD^2^, Chao Yan, PhD^2^, Weiyi Xia, PhD^2^, Congning Ni, MS^1^, Ellen Wright Clayton, MD, JD^3,4,5^, Yevgeniy Vorobeychik, PhD^6^, Murat Kantarcioglu, PhD^7^, Bradley A. Malin, PhD^1,2,8^

^1^Department of Computer Science, Vanderbilt University, Nashville, TN;

^2^Department of Biomedical Informatics, Vanderbilt University Medical Center, Nashville, TN;

^3^School of Law, Vanderbilt University, Nashville, TN;

^4^Department of Pediatrics, Vanderbilt University Medical Center, Nashville, TN;

^5^Department of Health Policy, Vanderbilt University Medical Center, Nashville, TN;

^6^Department of Computer Science and Engineering, Washington University in St. Louis, St. Louis, MO;

^7^Department of Computer Science, University of Texas at Dallas, Richardson, TX;

^8^Department of Biostatistics, Vanderbilt University Medical Center, Nashville, TN

Corresponding Author:

Bradley A. Malin, PhD

2525 West End Ave. Suite 1030, Nashville, TN, 37203

Department of Biomedical Informatics, Vanderbilt University Medical Center

Email: b.malin@vumc.org

## Section A. Topic Modeling

Figure 1 illustrates the change in coherence score as a function of the number of topics. We selected 10 as the number of topics, as it achieved the highest coherence score (0.391).

Figure 2 displays the distribution of the three post types and 10 topics in a 2D t-SNE scatterplot. Instead of showing all the posts in one figure, we selected 10% (1,587/15,596) of the most relevant posts to their dominant topics (the topic with the largest proportion). All the selected posts achieved a relevance score (the relevance between the post and its dominant topic) higher than 0.155. In general, the 10 clusters in Figure 8 are well separated, but cluster T_5_ (rhombus-shaped) and cluster T_9_ (pentagon-shaped) were close to each other, which suggests these two topics have a high degree of similarity.


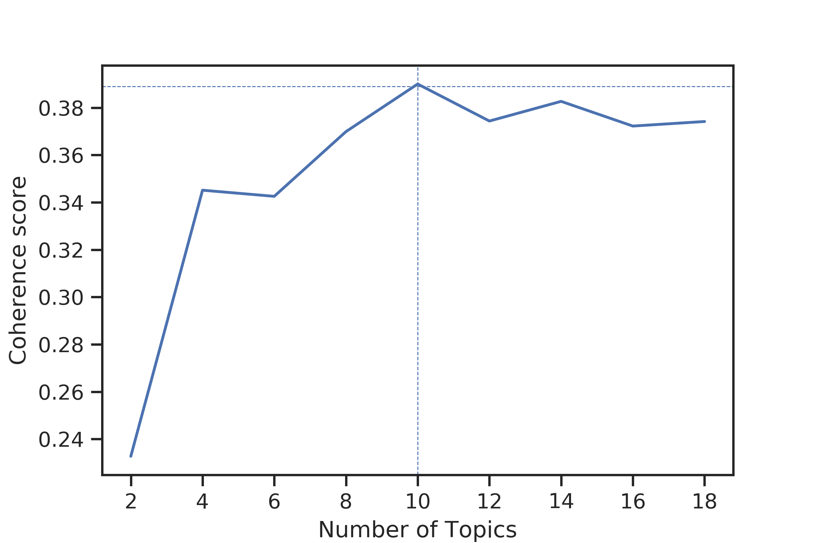


**Figure 1. Coherence score as a function of the number of topics.**


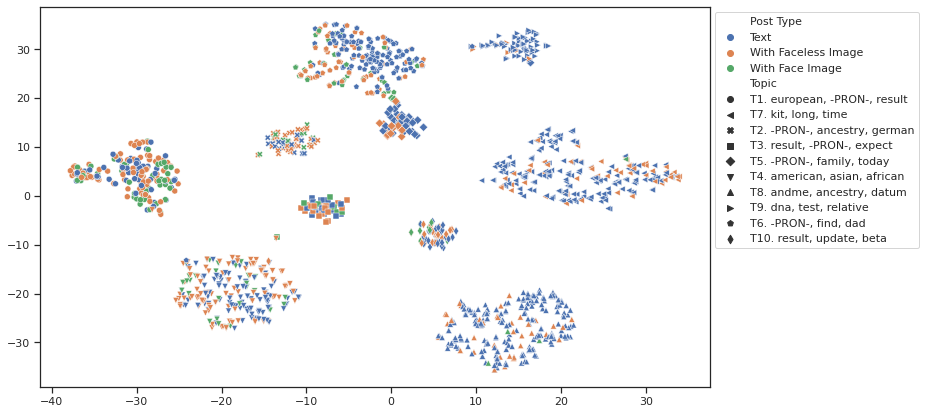


**Figure 2. t-SNE clustering result of 1,587 selected posts in 10 topics, the markers represent posts. The relevance between selected posts and their dominant topic is greater than 0.155.**

## Section B.

## Boxplots of the Entire Dataset


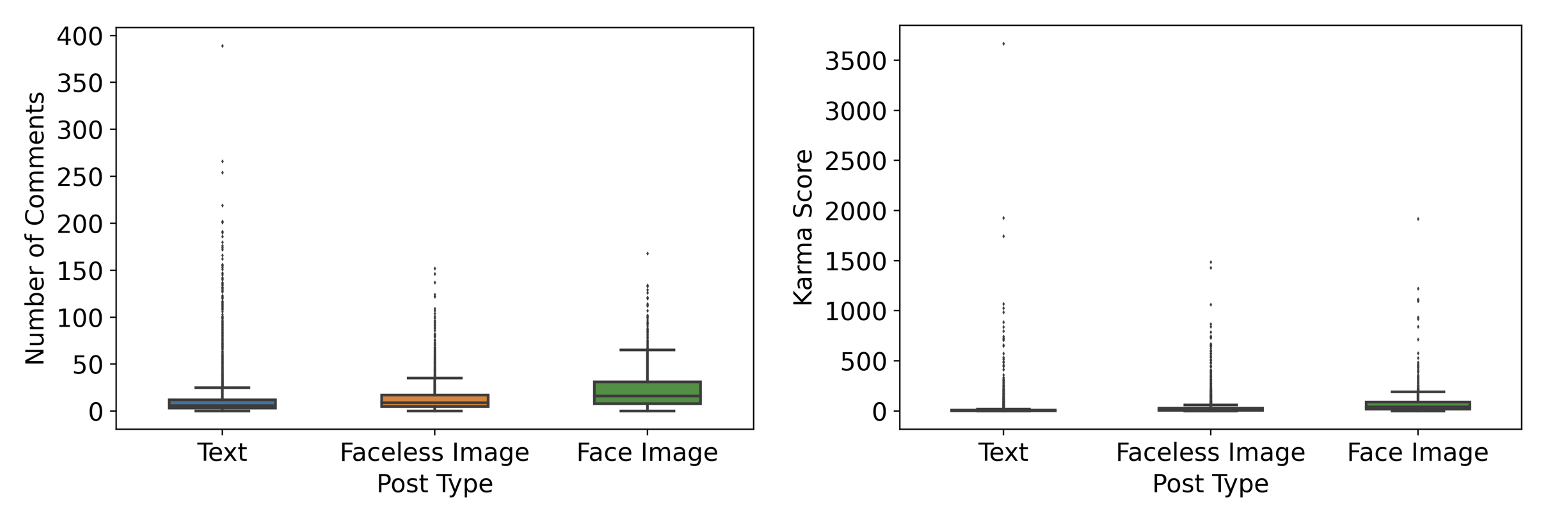


**Figure 3. Attention for three types of posts of entire data: number of comments per post (left) and Karma score per post (right).**


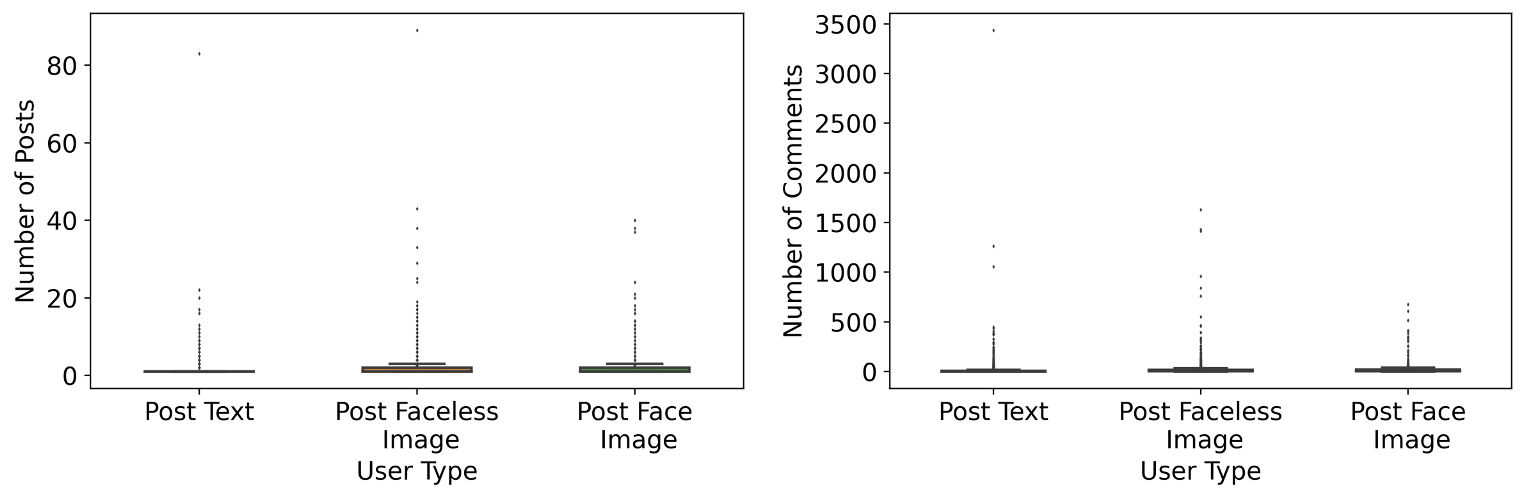


**Figure 4. Number of posts per user (left) and number of comments per user (right) for users who post 1) text only, 2) a faceless image, and 3) a face image.**
